# (*E*)-Ethyl 3-(2-fluoro­anilino)-2-(4-methoxy­phen­yl)acrylate

**DOI:** 10.1107/S160053680800175X

**Published:** 2008-01-23

**Authors:** Yi Zheng, Zhu-Ping Xiao, Kai-Rui Wang, Hai-Liang Zhu

**Affiliations:** a Institute of Functional Biomolecules, State Key Laboratory of Pharmaceutical Biotechnology, Nanjing University, Nanjing 210093, People’s Republic of China

## Abstract

The title compound, C_18_H_18_FNO_3_, consists of three individually planar subunits, namely two substituted benzene rings and one amino­acrylate group. The dihedral angle between the two benzene rings is 47.48 (8)°. The amino­acrylate group forms dihedral angles of 57.95 (7) and 11.27 (6)° with the methoxy­phenyl and fluorophenyl rings, respectively.

## Related literature

For related literature, see: Xiao *et al.* (2007 ▶, 2008 ▶).
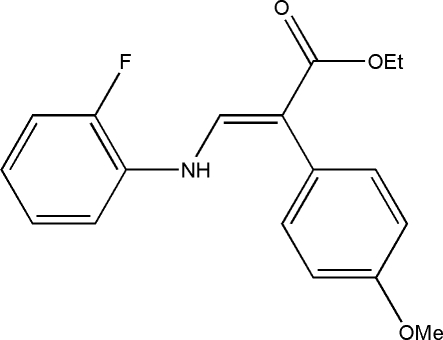


## Experimental

### 

#### Crystal data

C_18_H_18_FNO_3_
*M* *_r_* = 315.33
Triclinic, 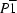

*a* = 6.3630 (13) Å
*b* = 9.4700 (19) Å
*c* = 13.981 (3) Å
α = 97.68 (3)°
β = 97.38 (3)°
γ = 95.40 (3)°
*V* = 822.7 (3) Å^3^
*Z* = 2
Mo *K*α radiation
μ = 0.09 mm^−1^
*T* = 298 (2) K
0.40 × 0.20 × 0.20 mm

#### Data collection

Enraf–Nonius CAD-4 diffractometer
Absorption correction: ψ scan (North *et al.*, 1968 ▶) *T*_min_ = 0.963, *T*_max_ = 0.981
3273 measured reflections
2980 independent reflections
1922 reflections with *I* > 2σ(*I*)
*R*_int_ = 0.044

#### Refinement

*R*[*F* ^2^ > 2σ(*F* ^2^)] = 0.058
*wR*(*F* ^2^) = 0.175
*S* = 1.09
2980 reflections
209 parameters
H atoms treated by a mixture of independent and constrained refinement
Δρ_max_ = 0.26 e Å^−3^
Δρ_min_ = −0.21 e Å^−3^

### 

Data collection: *CAD-4 Software* (Enraf–Nonius, 1989 ▶); cell refinement: *CAD-4 Software*; data reduction: *XCAD4* (Harms & Wocadlo, 1995 ▶); program(s) used to solve structure: *SHELXS97* (Sheldrick, 2008 ▶); program(s) used to refine structure: *SHELXL97* (Sheldrick, 2008 ▶); molecular graphics: *SHELXTL* (Sheldrick, 2008 ▶); software used to prepare material for publication: *SHELXTL*.

## Supplementary Material

Crystal structure: contains datablocks global, I. DOI: 10.1107/S160053680800175X/pv2066sup1.cif

Structure factors: contains datablocks I. DOI: 10.1107/S160053680800175X/pv2066Isup2.hkl

Additional supplementary materials:  crystallographic information; 3D view; checkCIF report

## Figures and Tables

**Table 1:** Hydrogen-bond geometry (Å, °)

*D*—H⋯*A*	*D*—H	H⋯*A*	*D*⋯*A*	*D*—H⋯*A*
N1—H1⋯O3^i^	0.91 (3)	2.65 (3)	3.256 (3)	125 (3)
C12—H12⋯O1^ii^	0.93	2.53	3.394 (3)	155
N1—H1⋯F1	0.91 (3)	2.26 (3)	2.654 (3)	102 (2)
C13—H13⋯O2	93.0	2.26	2.649 (3)	104

Symmetry codes: (i) 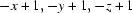; (ii) 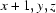.

**Fig. 1.**
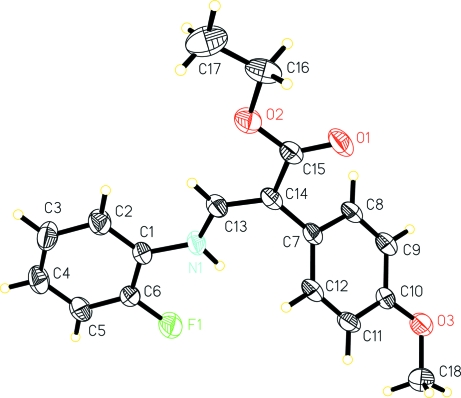
Molecular structure of the title compound, showing the atom-numbering scheme. Displacement ellipsoids are drawn at the 30% probability level.

## supplementary crystallographic information

### Comment

An enamine, a tautomer of a Schiff base, shows a high similarity to the corresponding Schiff base in chemical structure which shows diverse biological activities. Our recent work affirmed that enamine, like Schiff base, exhibited high antibacterial activity (Xiao *et al.*, 2007; Xiao *et al.*, 2008). We herein report the crystal structure of the title compound, (I), an enamine.

As shown in Fig. 1, (I) is structurally divided into three subunits, and each moiety forms a plane, namely, C1 to C6 forms a plane with the mean deviation of 0.0037 Å, defined as plane I; C7 to C12 forms a plane with the mean deviation of 0.0028 Å, defined as plane II; N1, C13, C14, C15, O1 and O2 is nearly coplanar with the mean deviation of 0.0119 Å, defined as plane III. Plane II and plane III make a dihedral angle with plane I of 47.48 (8) and 11.27 (6) °, and the dihedral angle between plane II and plane III is 57.95 (7) °. The bond distance C13—C14 (1.351 (4) Å) falls in the range of a typical double bond, and C13—N1 bond (1.340 (4) Å) is shorter than the standard C—N single bond (1.48 Å), but longer than a C—N double bond (1.28 Å). This clearly indicates that the p orbital of N1 seems to be conjugated with the π molecular orbital of C13—C14 double bond. All other double bonds and single bonds in the molecule fall in normal range of bond lengths. The structure is stabilized by intramolecular interactions involving rather weak hydrogen bonds of the types N—H···O and C—H···0 as well as intermolecular interactions amino-H···F and C13—H···O2; details of hydrogen-bond geometry are given in Table 1.

### Experimental

Equimolar quantities (6 mmol) of ethyl 2-(4-methoxyphenyl)-3- oxopropanoate (1.33 g) and 2-fluorobenzenamine (0.67 g) in absolute alcohol (18 ml) were heated at 344–354 K for 2 h. The excess solvent was removed under reduced pressure. The residue was purified by a flash chromatography with EtOAc-petrolum ether to afford two fractions. The first fraction gave a *Z*-isomer, and the second fraction, after partial solvent evaporated, furnished colorless blocks of (I) suitable for single-crystal structure determination.

### Refinement

The H atom bonded to N1 was located in a difference Fourier map. All other H atoms were placed in geometrically idealized positions and constrained to ride on their parent atoms with C—H = 0.93, 0.96 and 0.97 Å for the aromatic, CH_3_ and CH_2_ type H atoms, respectively. *U*_iso_ = 1.2*U*_eq_(parent atoms) were assigned for amino, aromatic and CH_2_ type H-atoms and 1.5*U*_eq_(parent atoms) for CH_3_ type H-atoms.

### Crystal data

**Table:**

C_18_H_18_FNO_3_	*Z* = 2
*M**_r_* = 315.33	*F*_000_ = 332
Triclinic, *P*1	*D*_x_ = 1.273 Mg m^−^^3^
*a* = 6.3630 (13) Å	Mo *K*α radiation λ = 0.71073 Å
*b* = 9.4700 (19) Å	Cell parameters from 1625 reflections
*c* = 13.981 (3) Å	θ = 1.5–25.0º
α = 97.68 (3)º	µ = 0.09 mm^−^^1^
β = 97.38 (3)º	*T* = 298 (2) K
γ = 95.40 (3)º	Block, colorless
*V* = 822.7 (3) Å^3^	0.40 × 0.20 × 0.20 mm

### Data collection

**Table:**

Enraf–Nonius CAD-4 diffractometer	2980 independent reflections
Radiation source: fine-focus sealed tube	1922 reflections with *I* > 2σ(*I*)
Monochromator: graphite	*R*_int_ = 0.044
*T* = 298(2) K	θ_max_ = 25.3º
ω/2θ scans	θ_min_ = 1.5º
Absorption correction: ψ scan(North *et al.*, 1968)	*h* = 0→7
*T*_min_ = 0.963, *T*_max_ = 0.981	*k* = −11→11
3273 measured reflections	*l* = −16→16

### Refinement

**Table:**

Refinement on *F*^2^	Hydrogen site location: inferred from neighbouring sites
Least-squares matrix: full	H atoms treated by a mixture of independent and constrained refinement
*R*[*F*^2^ > 2σ(*F*^2^)] = 0.058	*w* = 1/[σ^2^(*F*_o_^2^) + (0.0677*P*)^2^ + 0.314*P*] where *P* = (*F*_o_^2^ + 2*F*_c_^2^)/3
*wR*(*F*^2^) = 0.175	(Δ/σ)_max_ < 0.001
*S* = 1.09	Δρ_max_ = 0.26 e Å^−^^3^
2980 reflections	Δρ_min_ = −0.21 e Å^−^^3^
209 parameters	Extinction correction: SHELXL97 (Sheldrick, 2008), Fc^*^=kFc[1+0.001xFc^2^λ^3^/sin(2θ)]^-1/4^
Primary atom site location: structure-invariant direct methods	Extinction coefficient: 0.107 (9)
Secondary atom site location: difference Fourier map	

### Special details

**Table:**

Geometry. All e.s.d.'s (except the e.s.d. in the dihedral angle between two l.s. planes) are estimated using the full covariance matrix. The cell e.s.d.'s are taken into account individually in the estimation of e.s.d.'s in distances, angles and torsion angles; correlations between e.s.d.'s in cell parameters are only used when they are defined by crystal symmetry. An approximate (isotropic) treatment of cell e.s.d.'s is used for estimating e.s.d.'s involving l.s. planes.
Refinement. Refinement of *F*^2^ against ALL reflections. The weighted *R*-factor *wR* and goodness of fit *S* are based on *F*^2^, conventional *R*-factors *R* are based on *F*, with *F* set to zero for negative *F*^2^. The threshold expression of *F*^2^ > σ(*F*^2^) is used only for calculating *R*-factors(gt) *etc*. and is not relevant to the choice of reflections for refinement. *R*-factors based on *F*^2^ are statistically about twice as large as those based on *F*, and *R*- factors based on ALL data will be even larger.

### Fractional atomic coordinates and isotropic or equivalent isotropic displacement parameters (Å^2^)

**Table:**

	*x*	*y*	*z*	*U*_iso_*/*U*_eq_	
O3	0.4465 (3)	0.2358 (2)	0.49646 (15)	0.0698 (6)	
O2	−0.0514 (3)	0.6582 (2)	0.10142 (15)	0.0714 (6)	
O1	−0.1278 (3)	0.4580 (2)	0.16366 (17)	0.0771 (7)	
F1	0.9092 (3)	0.8076 (2)	0.36459 (16)	0.090	
C14	0.2001 (4)	0.6001 (3)	0.2237 (2)	0.0519 (7)	
C7	0.2661 (4)	0.5043 (3)	0.29442 (19)	0.0490 (6)	
C12	0.4551 (4)	0.4418 (3)	0.2923 (2)	0.0555 (7)	
H12	0.5395	0.4604	0.2448	0.067*	
N1	0.5153 (4)	0.7686 (3)	0.26886 (18)	0.0571 (6)	
C9	0.2078 (4)	0.3844 (3)	0.4321 (2)	0.0566 (7)	
H9	0.1244	0.3656	0.4799	0.068*	
C15	−0.0076 (4)	0.5629 (3)	0.1627 (2)	0.0557 (7)	
C1	0.6532 (4)	0.8864 (3)	0.25557 (19)	0.0508 (7)	
C10	0.3977 (4)	0.3237 (3)	0.4281 (2)	0.0507 (7)	
C8	0.1423 (4)	0.4720 (3)	0.3660 (2)	0.0533 (7)	
H8	0.0137	0.5105	0.3689	0.064*	
C13	0.3239 (4)	0.7195 (3)	0.2132 (2)	0.0543 (7)	
H13	0.2752	0.7717	0.1646	0.065*	
C5	1.0048 (5)	1.0171 (3)	0.2990 (3)	0.0709 (9)	
H5	1.1400	1.0267	0.3351	0.085*	
C11	0.5217 (4)	0.3533 (3)	0.3579 (2)	0.0589 (8)	
H11	0.6496	0.3139	0.3549	0.071*	
C6	0.8571 (4)	0.9063 (3)	0.3057 (2)	0.0580 (7)	
C2	0.6015 (5)	0.9858 (3)	0.1961 (2)	0.0691 (9)	
H2	0.4652	0.9776	0.1612	0.083*	
C4	0.9513 (5)	1.1144 (3)	0.2383 (2)	0.0738 (9)	
H4	1.0502	1.1902	0.2319	0.089*	
C3	0.7505 (6)	1.0982 (3)	0.1874 (3)	0.0780 (10)	
H3	0.7130	1.1638	0.1461	0.094*	
C18	0.6409 (5)	0.1735 (4)	0.4950 (3)	0.0800 (10)	
H18A	0.6384	0.1153	0.4330	0.120*	
H18B	0.6575	0.1151	0.5459	0.120*	
H18C	0.7581	0.2480	0.5050	0.120*	
C16	−0.2489 (5)	0.6276 (4)	0.0363 (3)	0.0823 (10)	
H16A	−0.3683	0.6266	0.0731	0.099*	
H16B	−0.2552	0.5344	−0.0031	0.099*	
C17	−0.2589 (7)	0.7410 (5)	−0.0268 (3)	0.1111 (15)	
H17A	−0.2568	0.8323	0.0128	0.167*	
H17B	−0.3881	0.7218	−0.0724	0.167*	
H17C	−0.1384	0.7425	−0.0616	0.167*	
H1	0.567 (5)	0.714 (4)	0.313 (3)	0.089 (11)*	

### Atomic displacement parameters (Å^2^)

**Table:**

	*U*^11^	*U*^22^	*U*^33^	*U*^12^	*U*^13^	*U*^23^
O3	0.0550 (12)	0.0796 (14)	0.0784 (14)	0.0045 (10)	0.0128 (10)	0.0239 (12)
O2	0.0526 (12)	0.0798 (15)	0.0765 (14)	−0.0031 (10)	−0.0079 (10)	0.0156 (12)
O1	0.0451 (12)	0.0827 (15)	0.0974 (17)	−0.0184 (11)	0.0068 (11)	0.0121 (12)
F1	0.054	0.096	0.121	−0.007	−0.008	0.048
C14	0.0367 (14)	0.0573 (16)	0.0594 (17)	−0.0039 (12)	0.0121 (12)	0.0024 (13)
C7	0.0382 (14)	0.0497 (15)	0.0562 (16)	−0.0067 (11)	0.0083 (12)	0.0041 (12)
C12	0.0409 (15)	0.0671 (18)	0.0596 (17)	−0.0034 (13)	0.0197 (13)	0.0080 (15)
N1	0.0470 (13)	0.0586 (15)	0.0633 (15)	−0.0110 (11)	−0.0012 (11)	0.0207 (12)
C9	0.0448 (16)	0.0693 (18)	0.0545 (17)	−0.0053 (13)	0.0166 (13)	0.0043 (14)
C15	0.0419 (15)	0.0633 (18)	0.0626 (18)	0.0018 (14)	0.0141 (13)	0.0089 (15)
C1	0.0462 (15)	0.0485 (15)	0.0563 (16)	−0.0034 (12)	0.0086 (12)	0.0084 (13)
C10	0.0418 (15)	0.0516 (15)	0.0560 (16)	−0.0051 (12)	0.0060 (12)	0.0072 (13)
C8	0.0366 (14)	0.0622 (17)	0.0602 (17)	−0.0019 (12)	0.0151 (12)	0.0037 (14)
C13	0.0442 (15)	0.0602 (17)	0.0574 (17)	−0.0025 (13)	0.0080 (13)	0.0098 (13)
C5	0.0482 (17)	0.0658 (19)	0.093 (2)	−0.0126 (15)	0.0047 (16)	0.0110 (18)
C11	0.0365 (14)	0.0654 (18)	0.074 (2)	0.0014 (13)	0.0133 (14)	0.0038 (15)
C6	0.0461 (16)	0.0542 (16)	0.075 (2)	0.0027 (13)	0.0055 (14)	0.0190 (15)
C2	0.0575 (19)	0.068 (2)	0.078 (2)	−0.0089 (15)	−0.0015 (16)	0.0184 (17)
C4	0.070 (2)	0.0584 (19)	0.088 (2)	−0.0192 (16)	0.0142 (18)	0.0082 (17)
C3	0.087 (3)	0.058 (2)	0.088 (2)	−0.0072 (17)	0.004 (2)	0.0278 (18)
C18	0.063 (2)	0.081 (2)	0.097 (3)	0.0125 (17)	0.0042 (18)	0.020 (2)
C16	0.0512 (19)	0.113 (3)	0.074 (2)	0.0140 (18)	−0.0053 (16)	−0.005 (2)
C17	0.112 (3)	0.133 (4)	0.085 (3)	0.041 (3)	−0.017 (2)	0.018 (3)

### Geometric parameters (Å, °)

**Table:**

O3—C10	1.374 (3)	C10—C11	1.376 (4)
O3—C18	1.421 (4)	C8—H8	0.9300
O2—C15	1.349 (3)	C13—H13	0.9300
O2—C16	1.435 (4)	C5—C6	1.363 (4)
O1—C15	1.198 (3)	C5—C4	1.370 (4)
F1—C6	1.362 (3)	C5—H5	0.9300
C14—C13	1.351 (4)	C11—H11	0.9300
C14—C15	1.463 (4)	C2—C3	1.386 (4)
C14—C7	1.478 (4)	C2—H2	0.9300
C7—C12	1.392 (4)	C4—C3	1.365 (5)
C7—C8	1.397 (4)	C4—H4	0.9300
C12—C11	1.377 (4)	C3—H3	0.9300
C12—H12	0.9300	C18—H18A	0.9600
N1—C13	1.364 (3)	C18—H18B	0.9600
N1—C1	1.400 (3)	C18—H18C	0.9600
N1—H1	0.91 (3)	C16—C17	1.479 (5)
C9—C8	1.374 (4)	C16—H16A	0.9700
C9—C10	1.391 (4)	C16—H16B	0.9700
C9—H9	0.9300	C17—H17A	0.9600
C1—C2	1.373 (4)	C17—H17B	0.9600
C1—C6	1.377 (4)	C17—H17C	0.9600
			
C10—O3—C18	117.1 (2)	C4—C5—H5	120.5
C15—O2—C16	116.8 (3)	C10—C11—C12	119.5 (3)
C13—C14—C15	119.2 (3)	C10—C11—H11	120.2
C13—C14—C7	122.7 (2)	C12—C11—H11	120.2
C15—C14—C7	118.2 (2)	F1—C6—C5	119.7 (3)
C12—C7—C8	117.1 (3)	F1—C6—C1	116.6 (2)
C12—C7—C14	121.1 (2)	C5—C6—C1	123.7 (3)
C8—C7—C14	121.8 (2)	C1—C2—C3	120.7 (3)
C11—C12—C7	122.4 (2)	C1—C2—H2	119.7
C11—C12—H12	118.8	C3—C2—H2	119.7
C7—C12—H12	118.8	C3—C4—C5	118.9 (3)
C13—N1—C1	125.7 (2)	C3—C4—H4	120.6
C13—N1—H1	117 (2)	C5—C4—H4	120.6
C1—N1—H1	116 (2)	C4—C3—C2	121.2 (3)
C8—C9—C10	120.5 (3)	C4—C3—H3	119.4
C8—C9—H9	119.8	C2—C3—H3	119.4
C10—C9—H9	119.8	O3—C18—H18A	109.5
O1—C15—O2	122.0 (3)	O3—C18—H18B	109.5
O1—C15—C14	125.0 (3)	H18A—C18—H18B	109.5
O2—C15—C14	113.0 (2)	O3—C18—H18C	109.5
C2—C1—C6	116.4 (2)	H18A—C18—H18C	109.5
C2—C1—N1	125.1 (3)	H18B—C18—H18C	109.5
C6—C1—N1	118.5 (2)	O2—C16—C17	107.6 (3)
O3—C10—C11	124.7 (3)	O2—C16—H16A	110.2
O3—C10—C9	115.8 (2)	C17—C16—H16A	110.2
C11—C10—C9	119.5 (3)	O2—C16—H16B	110.2
C9—C8—C7	121.0 (3)	C17—C16—H16B	110.2
C9—C8—H8	119.5	H16A—C16—H16B	108.5
C7—C8—H8	119.5	C16—C17—H17A	109.5
C14—C13—N1	124.4 (3)	C16—C17—H17B	109.5
C14—C13—H13	117.8	H17A—C17—H17B	109.5
N1—C13—H13	117.8	C16—C17—H17C	109.5
C6—C5—C4	119.1 (3)	H17A—C17—H17C	109.5
C6—C5—H5	120.5	H17B—C17—H17C	109.5
			
C13—C14—C7—C12	−56.8 (4)	C14—C7—C8—C9	−179.0 (2)
C15—C14—C7—C12	122.9 (3)	C15—C14—C13—N1	176.7 (3)
C13—C14—C7—C8	123.3 (3)	C7—C14—C13—N1	−3.5 (4)
C15—C14—C7—C8	−57.0 (3)	C1—N1—C13—C14	175.6 (3)
C8—C7—C12—C11	−0.8 (4)	O3—C10—C11—C12	179.2 (2)
C14—C7—C12—C11	179.3 (2)	C9—C10—C11—C12	−0.5 (4)
C16—O2—C15—O1	−0.6 (4)	C7—C12—C11—C10	0.5 (4)
C16—O2—C15—C14	177.9 (2)	C4—C5—C6—F1	179.0 (3)
C13—C14—C15—O1	177.8 (3)	C4—C5—C6—C1	−0.9 (5)
C7—C14—C15—O1	−2.0 (4)	C2—C1—C6—F1	−179.8 (3)
C13—C14—C15—O2	−0.6 (4)	N1—C1—C6—F1	0.4 (4)
C7—C14—C15—O2	179.7 (2)	C2—C1—C6—C5	0.1 (5)
C13—N1—C1—C2	12.3 (5)	N1—C1—C6—C5	−179.7 (3)
C13—N1—C1—C6	−168.0 (3)	C6—C1—C2—C3	0.7 (5)
C18—O3—C10—C11	1.1 (4)	N1—C1—C2—C3	−179.5 (3)
C18—O3—C10—C9	−179.3 (3)	C6—C5—C4—C3	0.9 (5)
C8—C9—C10—O3	−178.9 (2)	C5—C4—C3—C2	−0.1 (5)
C8—C9—C10—C11	0.8 (4)	C1—C2—C3—C4	−0.8 (5)
C10—C9—C8—C7	−1.1 (4)	C15—O2—C16—C17	−177.3 (3)
C12—C7—C8—C9	1.1 (4)		

### Hydrogen-bond geometry (Å, °)

**Table:**

*D*—H···*A*	*D*—H	H···*A*	*D*···*A*	*D*—H···*A*
N1—H1···O3^i^	0.91 (3)	2.65 (3)	3.256 (3)	125 (3)
C12—H12···O1^ii^	0.93	2.53	3.394 (3)	155
N1—H1···F1	0.91 (3)	2.26 (3)	2.654 (3)	102 (2)
C13—H13···O2	93.0	2.26	2.649 (3)	104

Symmetry codes: (i) −*x*+1, −*y*+1, −*z*+1; (ii) *x*+1, *y*, *z*.
